# The immune receptor XA21 causes semi-male sterility and grain loss in rice

**DOI:** 10.3389/fpls.2025.1673821

**Published:** 2025-11-10

**Authors:** Beatriz de Toledo Franceschi, Satyam Vergish, Yatendra Singh, Jian-Liang Li, Shamsunnaher, Gao-Lu Ding, Sixue Chen, Wen-Yuan Song

**Affiliations:** 1 Department of Plant Pathology, Institute of Food and Agricultural Sciences (IFAS), University of Florida, Gainesville, FL, United States; 2 Department of Biology, University of Mississippi, Oxford, MS, United States; 3 Integrative Bioinformatics, National Institute of Environmental Health Sciences, National Institutes of Health, Research Triangle Park, Durham, NC, United States

**Keywords:** fertility defect, anther dehiscence, jasmonate signaling, disease resistance, innate immune receptor, *Oryza sativa*

## Abstract

As part of an armory against pathogens, plants carry resistance (*R*) genes despite the fitness costs they can incur. While these detrimental effects have been associated with the presence and interactions of numerous *R* genes in various plant species, molecular models do not exist for the mechanisms underlying *R* gene-mediated fitness costs. The rice *R* gene *Xa21*, encoding a cell-surface immune receptor, specifies robust resistance to *Xanthomonas oryzae* pv. *oryzae*. Here, we demonstrate that *Xa21* expression causes drastic fertility defects, including reduced pollen viability, impaired anther dehiscence, and severe grain loss, at a low temperature (24°C) and in a dose-dependent manner. Under such growth conditions, *Xa21* plants displayed abundant accumulation of reactive oxygen species in their anthers and decreased expression of genes related to jasmonate biosynthesis, signaling, and response in their spikelets during anthesis. Consequently, jasmonate contents in XA21 spikelets were lower than those in the control. The exogenous application of methyl jasmonate largely rescued the anther dehiscence of *Xa21* plants. Given the key roles of lipid-derived jasmonates in stamen development and maturation in plants, our findings link *R* gene expression, jasmonic acid (JA) signaling, and fertility defects; identify temperature as an environmental factor influencing the range of *R* gene functions; and explain the abundant accumulation of 17 transposable-like elements previously observed in the *Xa21* locus.

## Introduction

Extensive research over the past three decades has led to the molecular characterization of diverse plant species of more than 300 disease resistance (*R*) genes that confer resistance to a variety of pathogens (Kourelis and van der Hoorn, 2018). Major families of characterized *R* genes include those encoding intracellular receptors containing nucleotide-binding and leucine-rich repeat (NLR) domains (Jones et al., 2016, 2024), cell-surface receptor-like proteins (RLPs), and receptor-like kinases (RLKs) (Thomas et al., 1998; Sun et al., 2004; Chen et al., 2006; Hu et al., 2017; Ercoli et al., 2022; Jones et al., 2024). Many *R* loci were identified in wild relatives during the breeding of disease-resistant crops (Jones et al., 2024). Often, *R* loci contain small gene families with alleles present in disease-susceptible individuals (Song et al., 1997; Parniske et al., 1997; Deng et al., 2017). In natural populations, both resistant and susceptible alleles may have coexisted for millions of years.

*R* genes are beneficial to host survival only when pathogen invasion occurs, while accumulating evidence shows that the presence of *R* genes can impose negative impacts on plant growth and reproduction in the absence of obvious infection (Kruger et al., 2002; Karasov et al., 2017; Calvo-Baltanas et al., 2020; Gao et al., 2024). This phenomenon is known as the fitness cost of resistance, which forms an important component of a more prevalent observation called growth-defense trade-offs (Huot et al., 2014; He et al., 2022). Pioneered by Vanderplank’s studies in the 1960s, fitness costs were first used to explain the lack of more durable partial resistance in potato varieties to late blight (caused by *Phytophthora infestans*) (Vanderplank, 1963; Brown and Rant, 2013). The barley recessive gene *mlo* confers strong, durable resistance to powdery mildew (Büschges et al., 1997) and has been widely deployed through plant breeding in Europe and other areas, but *mlo* resistance incurs a 5%–15% grain loss, causes necrotic leaf spotting, and increases susceptibility to other diseases (Jørgensen, 1992; Brown and Rant, 2013). Likewise, transgenic *Arabidopsis* plants harboring *RPM1* or *RPS5* (two *NLR*-type *R* genes countering *Pseudomonas* bacteria) produce 5%–10% fewer seeds than the susceptible controls in field trials (Tian et al., 2003; Karasov et al., 2014). Such costs of resistance have likely prevented fixation of *R* alleles within populations. Similarly, yield reduction has been observed in rice cultivars carrying either the *NLR* gene *Pi-ta* (a yield penalty of 12%) or the pyramided *NLR* genes *Pib*, *Pi25*, and *Pi54*, which specify resistance against rice blast disease caused by the fungal pathogen *Magnaporthe oryzae* (Jia et al., 2004; Dean et al., 2012; Wang et al., 2015; Ning et al., 2017; Peng et al., 2021; Tan et al., 2023). A large-scale genome-wide association study of 1,495 hybrid and parental rice lines revealed a correlation between high yields and alleles responsible for susceptibility to blight and blast diseases, reflecting a trade-off between yield performance and disease resistance to both bacteria and fungi in this agronomically important crop (Huang et al., 2015). Despite the broad importance of disease resistance in both evolution and agriculture, little is known about the underlying physiological and molecular mechanisms behind *R* gene-mediated fitness costs, particularly those leading to defective seed development.

Anther dehiscence is the last stage of stamen maturation in flowering plants and enables the release of mature pollen grains from the opened anther. This key step in pollination influences subsequent seed set (Wilson et al., 2011). Jasmonic acid (JA) and its derivatives (collectively called jasmonates) are required for stamen maturation and reproductive development, although JA signaling generally plays a negative role in vegetative growth and acts antagonistically with growth-promoting hormones to modulate growth-defense conflicts in plants (Wilson et al., 2011; Yang et al., 2012; Huot et al., 2014; He et al., 2022; Dhakarey et al., 2016; Acosta and Przybyl, 2019). Mutation of genes involved in JA biosynthesis and perception in *Arabidopsis* and rice often leads to male sterility, with deficiencies in pollen viability and anther dehiscence (McConn and Browse, 1996; Xie et al., 1998; Sanders et al., 2000; Stintzi and Browse, 2000; Ishiguro et al., 2001; Park et al., 2002; Riemann et al., 2003; Schilmiller et al., 2007; Nguyen et al., 2023). Although the pathways for JA biosynthesis and signaling have largely been elucidated in model species, our understanding of the regulatory networks controlling JA content and JA response in stamen is far from complete.

The rice *Xa21* gene confers resistance to the Gram-negative bacterium *Xanthomonas oryzae* pv. *oryzae* (*Xoo*) that causes bacterial leaf blight disease (Khush et al., 1990; Song et al., 1995). *Xa21* was originally isolated from a Mali accession of the African wild rice species *longistaminata*, which differs from many rice varieties in that it is perennial, its flowers are partially self-incompatible, and it can propagate asexually through its rhizomes (Khush et al., 1990; Tong et al., 2023). *Xa21* encodes an RLK protein (XA21) that is mainly localized on the plasma membrane and the endoplasmic reticulum of rice cells (Park et al., 2010a; Chen et al., 2010). XA21 binds to the sulfated *Xoo* peptide RaxX (RaxX-sY, required for activation of XA21-mediated immunity X, tyrosine-sulfated) that is homologous to phytohormones in the plant Peptide-containing Sulfated tYrosine (PSY) family, with eight members (OsPSY1–8) in rice (Pruitt et al., 2015, 2017; Luu et al., 2019). The activation of XA21 by RaxX-sY triggers various defense responses, including the production of reactive oxygen species (ROS) (Pruitt et al., 2015; Chen et al., 2021).

Rice is a tropical/subtropical crop that produces grains normally between 22 °C and 28 °C (Su et al., 2023). XA21-mediated resistance is dose-dependent (Zhang et al., 2024) and can be primed using a low-temperature treatment of 23 °C to 27°C (Chen et al., 2019). Under low-temperature conditions, the steady-state level of the XA21 protein is not significantly affected. We have recently reported that the XA21 protein can be cleaved by a spikelet-expressed rhomboid protease, OsRBL3b (Vergish et al., 2025). The elevated accumulation of XA21 caused by OsRBL3b mutations coincides with male sterility and yield reduction. In this article, we demonstrate that *Xa21* in the wild-type *OsRBL3b* background impairs anther dehiscence, decreases pollen viability, and reduces grain set at a lower temperature. Moreover, our data reveal a function of the immune receptor in negatively regulating JA levels and JA response and in positively modulating ROS production in reproductive tissues of rice.

## Materials and methods

### Plant materials and growth conditions

Transgenic lines used in this study were in either the *Oryza sativa* L. ssp. *japonica* cv. Taipei 309 (TP309) or cv. Kitaake (Kitaake) background. Seed germination on half-strength Murashige and Skoog medium supplemented with 30 g/L sucrose and 50 μg/mL hygromycin and growth in a greenhouse in the subtropical climate of Gainesville, FL, USA (29°39′55″N, 82°20′10″W) were described previously (Shamsunnaher et al., 2020).

### Plasmid construction and rice transformation

A 2,204-bp promoter fragment of the *Xa21* gene was PCR-amplified with the Xa21Pro-1/Xa21Pro-2 primers and cloned into the binary vector pCmH-GUS using *Hin*dIII–*Bam*HI. The resulting construct (*Xa21pro:GUS*) was transformed into rice cultivar TP309 using *Agrobacterium*-mediated transformation according to the procedure described previously (Vergish et al., 2025).

### Plant fertility, pollen viability, and anther dehiscence assays

Plants were first grown to the early booting stage and then moved to a temperature-controlled growth chamber [under LED light (200 μmol m^−2^ s^−1^) with a 13-h light (24°C)/11-h dark (21°C) photoperiod and 70% relative humidity] without pre-acclimation treatment.

Plant fertility was measured based on the number of filled and empty grains within the two uppermost panicles from at least five plants per line. To determine pollen viability, approximately 10 anthers from five plants per line were randomly harvested and squashed in a centrifuge tube. Five replicates per line were prepared. After staining with 1% (w/v) iodine–potassium iodide (I_2_^−^KI), the released pollen grains were visualized using a BX43 LED Fluorescence microscope (Olympus, Breinigsville, PA, USA). To determine anther dehiscence/indehiscence, at least 100 spikelets per line were harvested 2 hours after anthesis (HAA) from five plants. The dissected spikelets were stained with I_2_^−^KI solution and visualized using the Olympus microscope to determine the number of pollen grains on the stigmas. Anther indehiscence of a spikelet was scored when I_2_^−^KI-stained pollen grains were found inside the anthers but not on the stigmas. Cross-sectioning of anthers was performed after paraffin embedding. Briefly, collected anthers were fixed in Dietrich’s Formalin Acetic Acid for 16 hours at room temperature. Samples were processed using a Leica tissue processor. After embedding in paraffin, samples were sectioned to 10 μm using a Rotary Microtome (HM 355 S) and visualized using the Olympus microscope as above.

### RNA-seq analysis

Twenty rice spikelets were harvested at anthesis from five plants per line and pooled to minimize individual variations. Three biological replicates were prepared. Total RNA was extracted using TRIzol reagent (Ambion, Austin, TX, USA) according to the manufacturer’s instructions. The RNA integrity (RIN) of each sample was assessed to ensure RIN values greater than eight. To enrich mRNA, poly(A) selection was performed using the KAPA mRNA HyperPrep kit (Roche, Indianapolis, IN, USA). The purified RNA was then used for RNA-seq library construction and paired-end (2 × 151 base read length) sequencing using the NovaSeq 6000 platform (Illumina, San Diego, CA, USA) at the Comprehensive Cancer Center (Monrovia, CA, USA). Approximately 60 million paired reads were generated per sample. Following trimming for adapters using the TrimGalore program (version 0.6.10), the cleaned reads were aligned to the *O. sativa* ‘Nipponbare’ reference genome (Kawahara et al., 2013) using HISAT2 (version v2.2.1) (Kim et al., 2015). Ambiguous reads that mapped to more than one region in the genome or those with a MAPQ score below 10 were removed using the SAMtools software (Genome Research Limited). Transcript quantification was performed using the Partek Genomics Suite (version 7.18) to obtain raw read counts and normalized read counts [reads per kilobase per million mapped reads (RPKM)] (Mortazavi et al., 2008). Differential gene expression was determined using generalized linear model approaches implemented in the Bioconductor package edgeR (McCarthy et al., 2012). The differentially expressed genes (DEGs) were assessed for significance based on the following criteria: absolute fold change of over 2 and false discovery rate (FDR) q-value below 0.05. Genes with a fold change greater than or equal to 2 were considered upregulated, whereas those with a fold change less than or equal to 2 were considered downregulated.

### RT-qPCR analysis

Total RNA was extracted using TRIzol reagent (Ambion) followed by purification using an RNAeasy MiniElute Cleanup Kit (Qiagen, Valencia, CA, USA) according to the manufacturer’s instructions. Single-stranded cDNA was synthesized with 2 μg total RNA using a QuantiTect Reverse Transcription Kit (Qiagen). qPCR was carried out using a LightCycler 480 System (Roche) according to the manufacturer’s instructions under the following conditions: 95°C, 2 min; 45 cycles of 95°C, 5 sec and 60°C, 20 sec, and 72°C, 5 min. The gene expression levels were calculated by the ΔΔCt method using the geometric mean of rice *UBIQUITIN 5* and *ACTIN* expression levels to normalize the data (Jain et al., 2018). Primers used for q-PCR are listed in **Supplementary Table 1**.

### LC–tandem mass spectrometry quantification of jasmonates

For JA quantification, spikelets that just opened were collected (stage 14 of anther development). Twenty spikelets (~160 mg, fresh weight) from five plants per line were transferred to a 2-mL screw cap tube (Cole-Parmer, Vernon Hills, IL, USA) and immediately frozen in liquid nitrogen. Four biological replicates/samples per line were performed. For each sample tube, a 6-mm stainless steel grinding ball (Cole-Parmer) was added. The sample was homogenized in a Spex^®^ HG-400 MiniG^®^ Homogenizer (Cole-Parmer) at 1,000 rpm for 5 min, with 1-min intervals. During grinding, liquid nitrogen was added to the sample trays to maintain the low temperature. After homogenization, internal standards (10 μL of 100 μM lidocaine and 10 μL of 100 μM 10-camphorsulfonic acid) were added to each sample. A serial extraction was performed with three pre-cooled solvents: extraction solvent I (acetonitrile:isopropanol:water, 3:3:2), extraction solvent II (acetonitrile:water, 1:1), and extraction solvent III (80% methanol). For each solvent, 800 μL was added, followed by 10 min of mixing by vortex and sonication in ice water for 6 min in 2-min intervals. After each extraction, samples were centrifuged at 15,000 × *g* for 10 min at 4°C, and the supernatants were collected. The three extracts were combined, lyophilized to dryness, and stored at −20 °C until further analysis. For liquid chromatography–tandem mass spectrometer (LC-MS/MS) analysis, 100 μL of 0.1% formic acid in water was added to each dried extract, followed by solubilization and centrifugation (15,000 × *g*, 4 °C, 10 min). The supernatants were lyophilized again and reconstituted in 25 μL of 0.1% formic acid in water. A final centrifugation step was performed under the same conditions, and the supernatants were transferred to LC–MS vials for analysis. The internal standards and authentic standards of JA and JA-isoleucine (JA-Ile) were purchased from Sigma (St. Louis, MO, USA).

Quantification analysis was performed using a microflow UPLC-ZenoTOF 7600 mass spectrometer (MS) (SCIEX, Toronto, ON, Canada), equipped with an OptiFlow™ Turbo V ion source. Chromatographic separation was achieved on a nanoEase™ M/Z Symmetry^®^ C18 column (5 μm, 300 μm i.d. × 50 mm, 100 Å) at a flow rate of 7 μL/min. The column chamber was maintained at 40°C, and the autosampler was maintained at 6°C. The injection volume for all the samples was 1 μL. The total run time was 10 min. The LC gradient consisted of mobile phase A (water with 0.1% formic acid) and mobile phase B (acetonitrile with 0.1% formic acid) and started at 90% A (0.0–0.5 min), decreased to 10% A (by 5 min), and, following a hold for 2 min, returned to 90% A (by 10 min). The eluent was introduced into the MS using the OptiFlow Turbo V source with a microflow probe under electrospray ionization (ESI) in positive mode. ESI source parameters were as follows: curtain gas, 35 psi; Collisionally Activated Dissociation (CAD) gas, 7 psi; GS1, 30 psi; GS2, 35 psi; and source temperature, 200°C. The MS/MS spectra for JA and JA-Ile were acquired using the function of multiple reaction monitoring high resolution (MRMhr) by including the [M + H]+ precursor ion. The precursor of JA at *m*/*z* 211.13 [M + H]+ yields product ions *m*/*z* 133.10 (quantifier) and 69.07 (qualifier) at a collision energy of 20 eV. The precursor of JA-Ile at *m*/*z* 324.21 [M + H]+ yields product ions *m*/*z* 151.11 (quantifier) and 86.09 (qualifier) at a collision energy of 20 eV. The declustering potential was set at 40 V, and the accumulation time was 0.05 ms for all MRMhr transitions. A five-point calibration curve (2.56, 12.8, 64, 320, and 1,600 pg/μL) was used for quantification. Data acquisition and processing were performed using the SciexOS 3.1 software (SCIEX, 2015).

### Dehiscence restoration by exogenous MeJA

To determine whether the dehiscence could be rescued, *Myc-Xa21^L^* and *Myc-Xa21^H^* plants were treated with exogenous methyl jasmonate (MeJA). Two panicles per plant (five plants of each line) were sprayed with either MeJA solution [200 μM MeJA (PhytoTech Labs, Lenexa, KS, USA) and 0.1% Tween] or mock solution (0.1% Tween) in the morning. At least 100 spikelets were harvested 2 HAA and dissected to determine anther dehiscence/indehiscence as described above.

### Histochemical GUS staining

GUS staining was carried out as described (Anbu and Arul, 2013). Freshly collected spikelets were immersed in GUS staining solution [100 mM sodium phosphate, pH 7.0, 10 mM EDTA, 0.1% (v/v) Triton X-100, 1 mM potassium ferrocyanide, 1 mM potassium ferricyanide, 20% (v/v) methanol, 2 mM X-Gluc], vacuum-infiltrated for 10 min, and incubated overnight at 37 °C. The stained samples were incubated in 100% (v/v) ethanol for 10 hours to remove chlorophyll.

### ROS assays

Staining with 3,3′-diaminobenzidine (DAB) was performed as described (Li et al., 2023). Freshly collected spikelets were immersed in DAB solution (1 mg/mL, pH 3.8) overnight and then boiled in ethanol for 10 min, followed by several washes in ethanol. Seven DAB-stained anthers per line were used. Approximate borders of DAB-stained areas in each anther were traced. The corresponding areas were measured in pixels using the image processing software Fiji/ImageJ (ImageJ, RRID: SCR_003070). Relative DAB-stained areas were calculated by dividing the number of DAB-stained pixels by the total number of anther pixels.

## Results

### XA21 drastically reduces grain set in a dose-dependent manner at a lower ambient temperature (24°C)

Since lower temperatures prime XA21 resistance to *Xoo* (Chen et al., 2019), we reasoned that low temperature may be sufficient to induce the XA21-dependent fertility defects even in the presence of the functional *OsRBL3b*. To test this hypothesis, we used two previously characterized transgenic lines (*Myc-Xa21^L^* and *Myc-Xa21^H^* in the background of TP309) carrying the same construct encoding an N-terminal c-Myc-tagged XA21 (Myc-XA21) expressed from its native promoter (Xu et al., 2006; Wang et al., 2006; Chen et al., 2019; Vergish et al., 2025). The Myc-XA21 protein accumulated to a higher level in line *Myc-Xa21^H^* than in line *Myc-Xa21^L^* in spikelets at 28°C (Vergish et al., 2025). We normally grow these plants in our outdoor greenhouse in Florida during the summer, with daytime temperatures ranging from 30 °C to 43°C.

**Figure 1:**
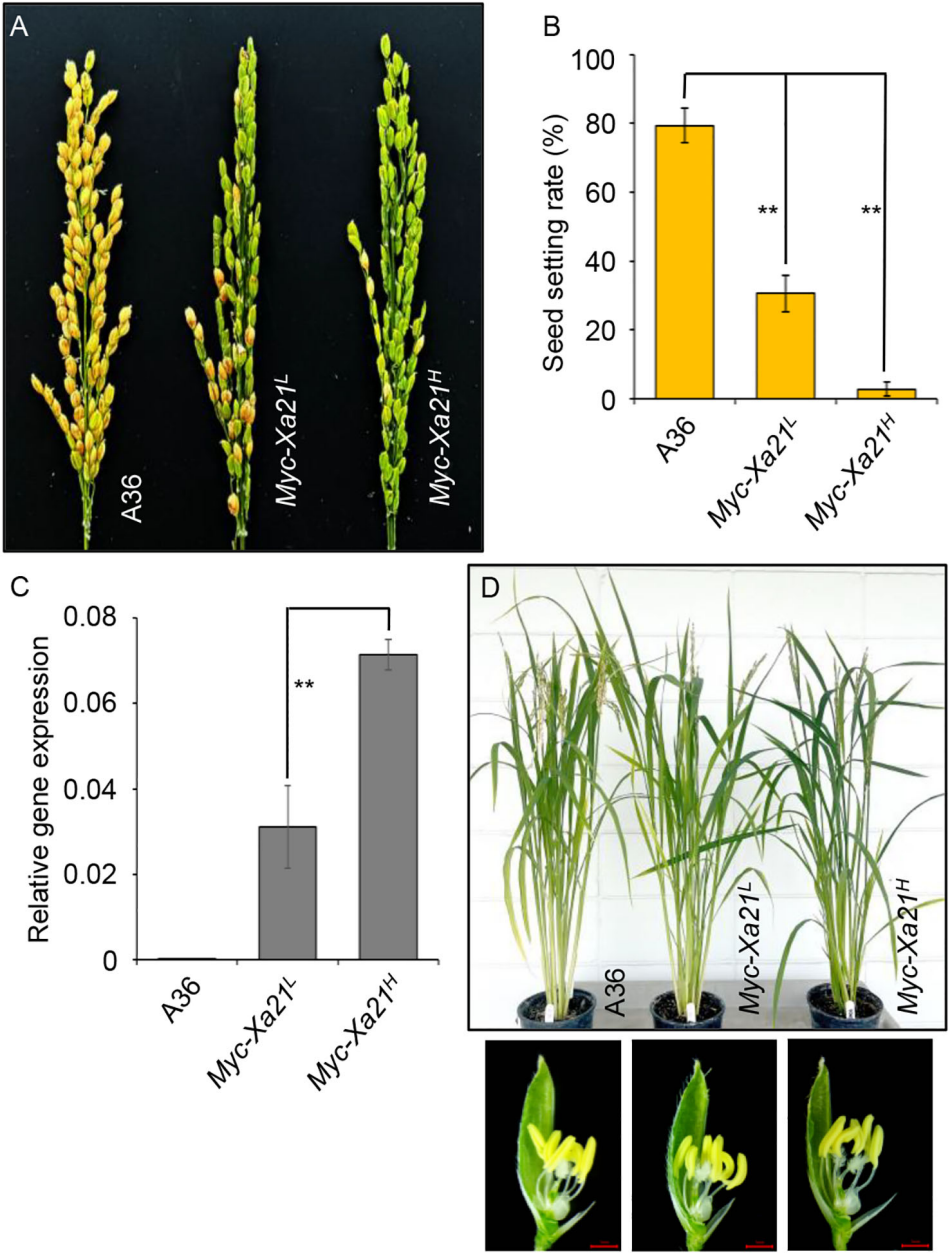
*Xa21* decreases grain set in rice at 24°C. **(A)** Panicle phenotypes of the rice lines A36 (empty vector control), *Myc-Xa21^L^*, and *Myc-Xa21^H^*. **(B)** Grain-setting rates of plants in **(A)** Values are shown as the mean ± SD from five independent plants with two panicles each. Statistical analyses were performed using Student’s *t*-test. Asterisks denote statistically significant differences (**p < 0.01). **(C)** Phenotypes of mature plants (*top*) and floral structures (*bottom*) of the indicated lines in **(A)** Scale bars, 1 mm. The experiments were repeated twice with similar results. **(D)** Relative transcript levels of the *Xa21* gene in the spikelets of the indicated lines grown at 24°C at anthesis. Results were normalized relative to levels of the mRNAs for *UBIQUITIN 5* and *ACTIN*. Values are means ± SD of three biological replicates, each with three technical replicates. Statistical analyses were performed using Student’s *t*-test. Asterisks denote statistically significant differences (**p < 0.01).

When grown at a lower temperature in a growth chamber [under LED light (200 μmol m^−2^ s^−1^) with a 13-h light (24°C)/11-h dark (21°C) photoperiod under 70% relative humidity, a growth condition labeled 24°C], these plants displayed drastically reduced grain set (**Figure 1A**) in the absence of the bacterial pathogen *Xoo* (*Xoo* is a quarantine pathogen in the USA). The average rates of grain set for the *Myc-Xa21^L^* and *Myc-Xa21^H^* plants were 30.52% and 2.71%, respectively, compared to 79.53% for the empty vector control line A36 (**Figure 1B**). These rates in *Xa21* plants were inversely correlated to the transcript levels of the *Xa21* gene in the spikelets at anthesis from plants grown at 24°C (**Figure 1C**). Apart from the fertility defect, *Xa21* plants appeared normal (**Figure 1D**).

When *Myc-Xa21* plants were grown in the greenhouse during the summer, no significant differences in grain set were observed (Vergish et al., 2025). Similarly, a significant reduction in grain set was also observed from the previously characterized transgenic line 20-1 (average rate of grain set: 62.70%), which expresses *Myc-Xa21* under its native promoter (Park et al., 2010b), relative to the wild-type control *O. sativa* ssp. *japonica* variety Kitaake (Kitaake, average rate of grain set: 86.01%) when grown at 24°C (**Supplementary Figure S1**). Compared to TP309, Kitaake is shorter in stature and has a shorter life cycle of approximately 9 weeks (Kunihiro et al., 1989; Kim et al., 2013). Together, these findings indicate that XA21 expression can induce a strong fertility defect in a dose- and temperature-dependent manner.

### XA21 plants display compromised pollen viability and anther dehiscence at 24°C

**Figure 2:**
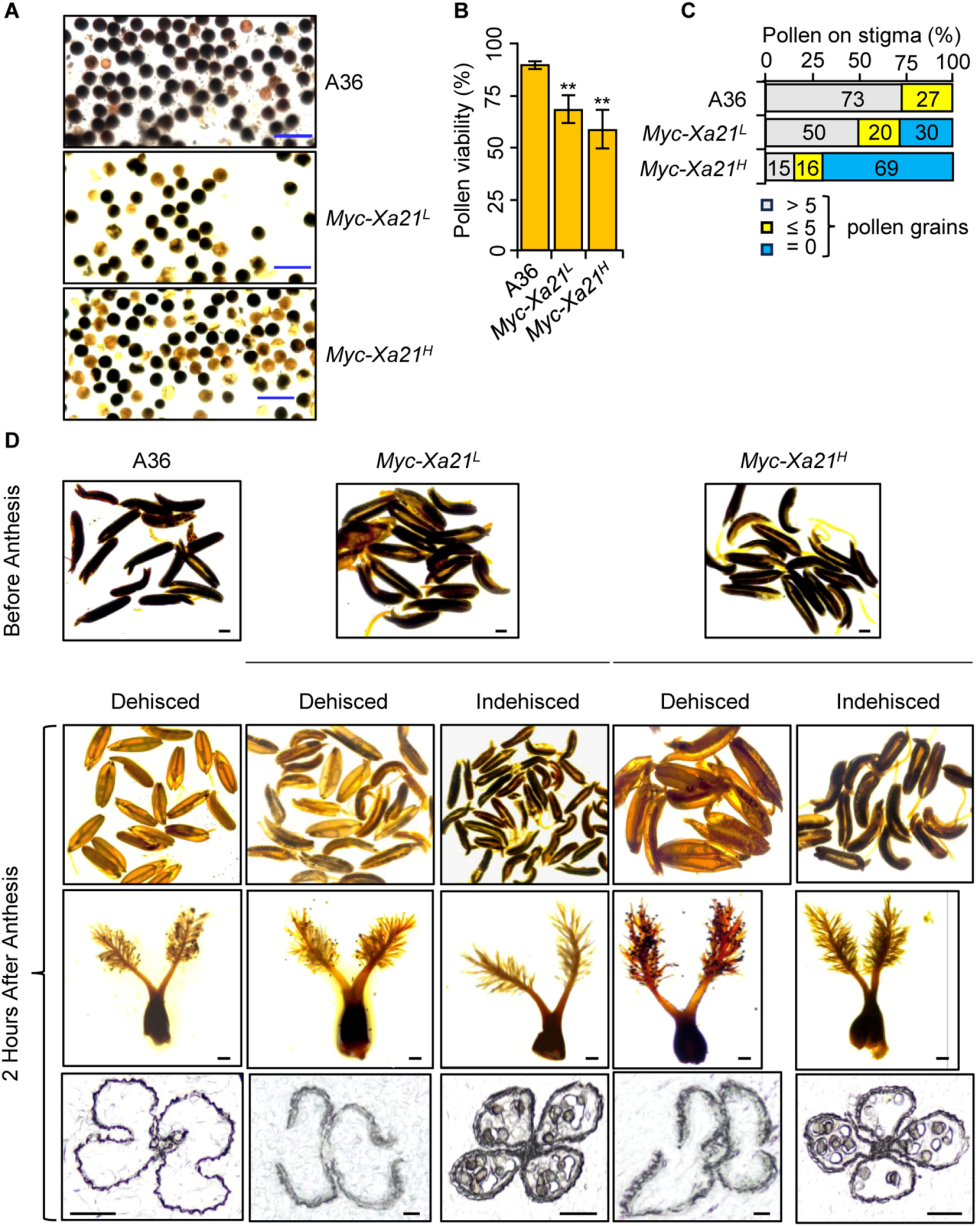
*Xa21* impairs pollen viability and anther dehiscence at 24°C. **(A, B)***Myc-Xa21* plants exhibit reduced starch accumulation in their pollen grains. I_2_^−^KI-stained pollen grains from the indicated lines **(A)**. Scale bars, 100 μM. Pollen viability was determined based on the number of stained pollen grains (dark color in **A**) relative to the total pollen counted **(B)**. Values are means ± SD, n = 5 (five independent samplings). Statistical analysis was performed using Student’s *t*-test. Asterisks indicate statistically significant differences (**p < 0.01). This experiment was repeated two times with similar results. **(C)** Pollen grains on the stigmas of the indicated lines after anthesis. The graph shows the distribution of dehiscent and indehiscent spikelets among 100 random flower samples chosen from five individual plants of each indicated line. **(D)** Presence or absence of pollen grains on the stigmas of the indicated lines after anthesis. (*Top 2 rows*) I_2_^−^KI staining of pollen grains in anthers of the indicated lines before and after anthesis. Scale bars, 500 μM. (*Third row*) Pollen grains on the stigmas of the indicated lines after anthesis. Scale bars, 200 μM. (*Bottom row*) Cross-sections of anthers from the indicated lines after anthesis. Scale bars, 100 μM.

Our recent study showed that *osrbl3b Myc-Xa21* mutants accumulate increased levels of the Myc-XA21 protein and exhibit pollen and dehiscence defects (Vergish et al., 2025). We examined whether XA21 expression affects pollen viability and anther dehiscence at 24°C. The *Myc-Xa21* plants produced lower levels of viable pollen grains than the control (A36), as evidenced by starch staining with iodine–potassium iodide (I_2_^−^KI) solution (**Figures 2A, B**).

To assess anther dehiscence, we first examined the presence of pollen grains (stained with I_2_^−^KI) in the anthers of *Myc-Xa21* and A36 plants before anthesis and 2 HAA. To confirm anther dehiscence, we further examined the presence of pollen grains on the stigmas and the breakage of anther walls 2 HAA. Among more than 100 randomly chosen spikelets, most anthers opened in A36 spikelets, and indehiscent ones were rare (**Figures 2C, D**). In contrast, 30%–69% of spikelets from the *Myc-Xa21^L^* and *Myc-Xa21^H^* lines were indehiscent 2 HAA. These data demonstrate that *Myc-Xa21* plants have a defect in anther dehiscence, which can partially explain the reduced grain set.

### XA21 downregulates the expression of a subset of JA-related genes at 24°C

**Figure 3:**
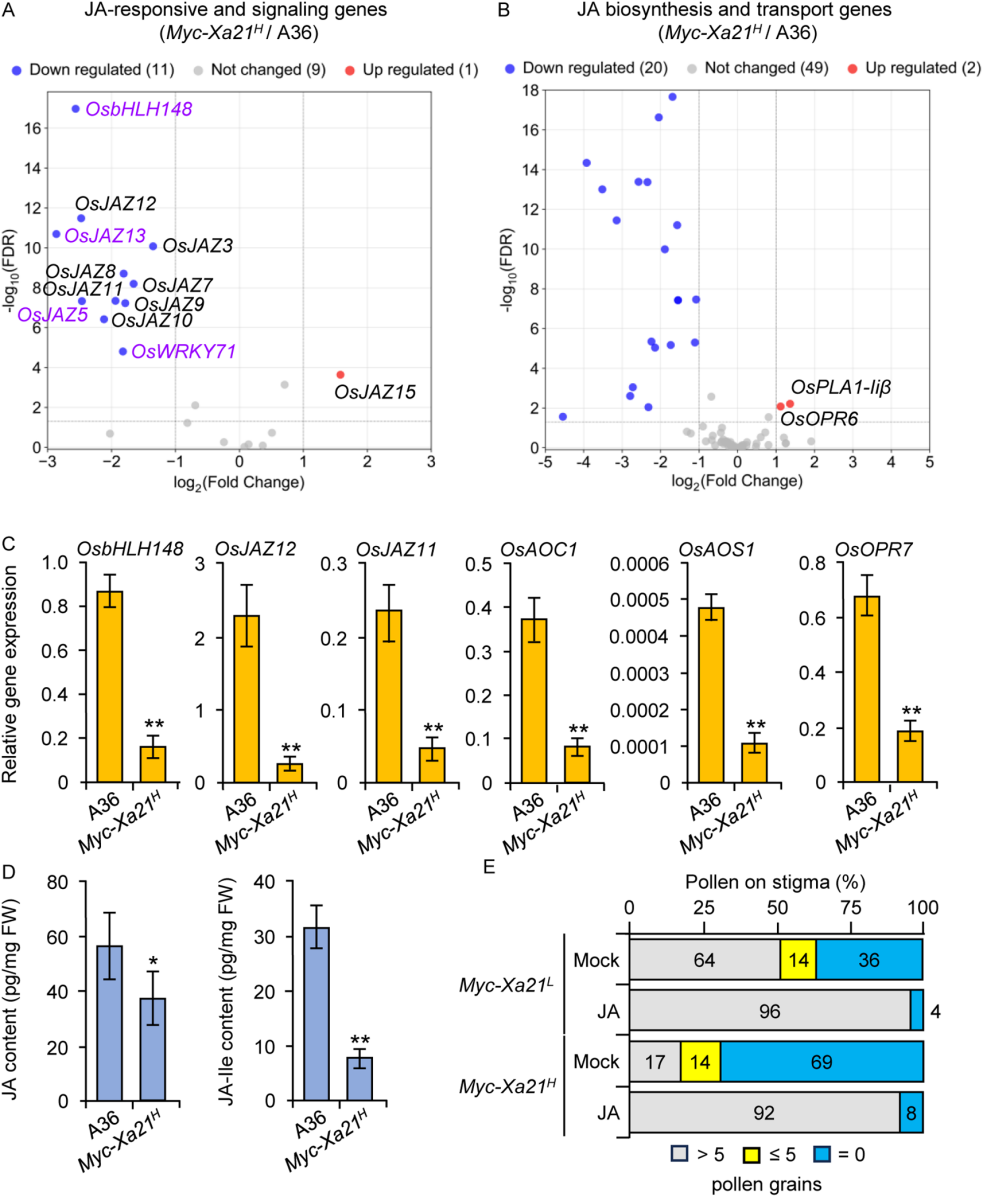
Downregulation of jasmonic acid (JA)-related genes in the indehiscent *Myc-Xa21^H^* spikelets relative to the dehiscent A36 spikelets at anthesis at 24°C. **(A)** RNA-seq volcano plot showing differentially expressed JA-responsive and JA-signaling genes. The genes highlighted in purple are also downregulated in the spikelets of *osrblb-b Myc-Xa21^H^* mutant during anthesis (Vergish et al., 2025). **(B)** RNA-seq volcano plot showing differentially expressed JA biosynthesis and transport genes. Downregulated genes in (**B**) are *OsPLA1α1*, *OsPLA1α3*, *OsPLA1β1*, *OsPLA1β2*, *OsLOX5*, *OsLOX6*, *OsLOX9*, *OsLOX11*, *OsLOX12*, *OsLOXL-2*, *OsAOS1*, *OsAOS2*, *OsAOS3*, *OsAOC1*, *OsOPR1*, *OsOPR7*, *OsJMT1*, *OsABCG1*, *OsABCG22*, and *OsABCG23*. **(C)** Relative transcript levels, quantified by RT-qPCR, of indicated JA-responsive and JA biosynthesis genes in **(A, B)** Results were normalized relative to transcript levels of *UBIQUITIN 5* and *ACTIN*. Values are means ± SD of three biological replicates, each with three technical replicates. Statistical analyses were performed using Student’s *t*-test. Asterisks denote significant differences (**p < 0.01). **(D)** Relative abundances of JA and JA-Ile, quantified by MS/MS, in the spikelets of the fertile A36 and the semi-sterile *Myc-Xa21^H^*. Values are means ± SD of four biological replicates. Statistical analyses were performed using Student’s *t*-test. Asterisks denote significant differences (**p < 0.01; *p < 0.05). A36 is a transgenic empty vector control. **(E)** Restoration of anther dehiscence of XA21 plants by exogenous JA treatment at 24°C. Mature panicles of indicated plants were sprayed with either 200 μM MeJA or mock solution. The graph shows the distribution of dehiscent and indehiscent spikelets among 100 random flower samples chosen from five plants of each indicated line at 2 hours after anthesis (HAA).

The sterile *osrbl3b-b Myc-Xa21^H^* mutant at higher temperatures shows the downregulation of JA-responsive and JA-signaling genes relative to the fertile line *Myc-Xa21^H^*, although no obvious enrichment of transcripts related to JA biosynthesis or transport is evident in the mutant (Vergish et al., 2025). To understand the mechanisms underlying the XA21-mediated fertility defects, we sequenced the transcripts in spikelets at anthesis from the temperature-sensitive *Myc-Xa21^H^* and the fertile A36 lines grown at 24°C. We identified a total of 4,792 DEGs between these two lines using an FDR value of <0.05 and log_2_FC cut-off criteria of >1 and <−1. The downregulated genes included 11 JA-responsive and JA-signaling genes in *Myc-Xa21^H^*, which encode nine JAZ transcriptional repressors (Chini et al., 2007; Thines et al., 2007; Ye et al., 2009) and the transcription factors OsbHLH148 (Seo et al., 2011) and OsWRKY71 (Liu et al., 2007) (**Figure 3A**). Among the 15 *JAZ* genes identified in the rice genome (Ye et al., 2009), only *OsJAZ15* was upregulated in *Myc-Xa21^H^* spikelets.

Interestingly, among the 70 genes related to JA biosynthesis and transport in the rice genome (Marla and Singh, 2012; Matsuda et al., 2012; Singh et al., 2012; Fukumoto et al., 2013; Riemann et al., 2013; Huang et al., 2014; Qi et al., 2016; Liu et al., 2017; Deepika and Singh, 2021), 20 were significantly downregulated (only two were upregulated) in the spikelets of *Myc-Xa21^H^* compared to those of the control (A36) (**Figure 3B**). The downregulated genes included the key JA biosynthetic genes *OsAOC1*, *OsAOS1*, and *OsOPR7* (Riemann et al., 2003, 2013; Hibara et al., 2016; Pak et al., 2021; Fan et al., 2023; Wang et al., 2024). The downregulation of six of the above genes was verified using qRT-PCR analysis (**Figure 3C**). Therefore, the XA21-mediated downregulation of JA-responsive genes may occur through the reduction of JA levels.

To test this, the abundance of JA and JA-Ile (the most bioactive form of jasmonates) was quantified in the spikelets of *Myc-Xa21^H^* and A36 plants grown at 24°C at anthesis using LC–MS/MS analysis. JA and JA-Ile were identified in all the samples using MS/MS spectral matching between authentic standards and metabolites from the samples (**Supplementary Figure S2**). This is level 1 structural identification, the most confident level based on the well-accepted Metabolomics Standards Initiative (Sumner et al., 2007). Based on the MS/MS fragmentation pattern of JA and JA-Ile, specific multiple reaction monitoring (MRM) transitions were selected for targeted metabolite quantification and validation. Relative peak areas were normalized using lidocaine as the internal standard, and then spikelet fresh weight was taken into account for quantitative analysis. As shown in **Figure 3D**, JA levels were significantly lower in *Myc-Xa21^H^* than in A36 spikelets, and JA-Ile was also significantly decreased in *Myc-Xa21^H^* relative to A36 spikelets.

To further test the above hypothesis, we sprayed the mature panicles of *Myc-Xa21^L^* and *Myc-Xa21^H^* plants with either 200 μM MeJA or a mock control. The dehiscence of male-sterile JA-related mutants can be restored by treatment with exogenous MeJA (Ishiguro et al., 2001; Song et al., 2018). Indeed, JA treatment largely restored the anther dehiscence of *Myc-Xa21* plants at 24°C (**Figure 3E**). Together, these findings support that the compromised anther dehiscence in *Myc-Xa21* plants may be due to a deficiency in JA levels within rice spikelets.

### *Xa21* is preferentially expressed in the anther filaments, veins, and rachillae of rice spikelets

**Figure 4:**
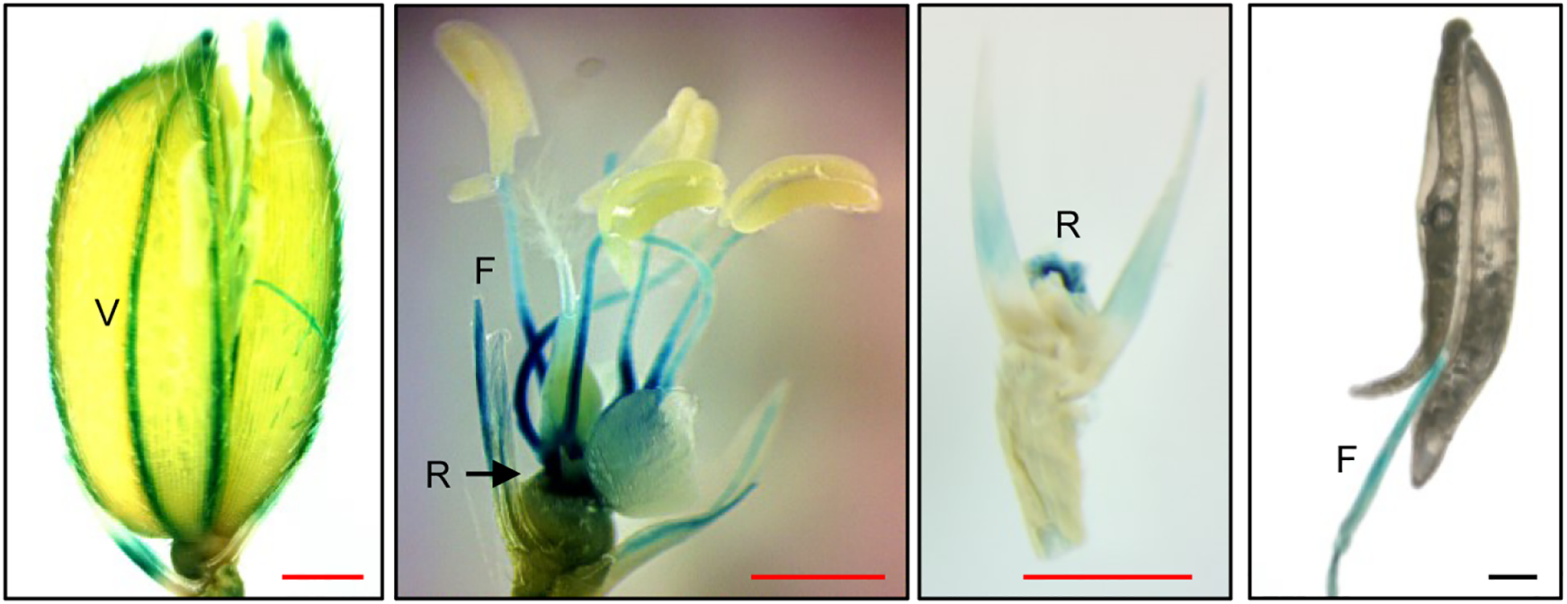
Histochemical analysis of GUS activity from *Xa21pro:GUS* reporter lines in spikelets. GUS activity was detectable in veins (V) of lemma and palea, in rachillae (R), and in anther filament (F) in the *Xa21pro:GUS* lines. Scale bars, 1 mm (red), 100 μm (black). Similar results were obtained from two independent (*Xa21pro:GUS*) lines.

To determine the expression sites of *Xa21* in spikelets, transgenic rice lines expressing the *uidA* (GUS reporter) gene were generated under the control of the *Xa21* promoter. This 2.0-kb promoter sequence is sufficient for directing *Xa21* functions (Song et al., 1995; Vergish et al., 2025). GUS assays detected strong activities in the anther filaments, the veins of the palea/lemma, and the rachillae of the spikelets (**Figure 4**). Notably, little GUS activity was observed in anthers.

### ROS levels increase in the anther of *Myc-Xa21* lines at anthesis at 24°C

**Figure 5:**
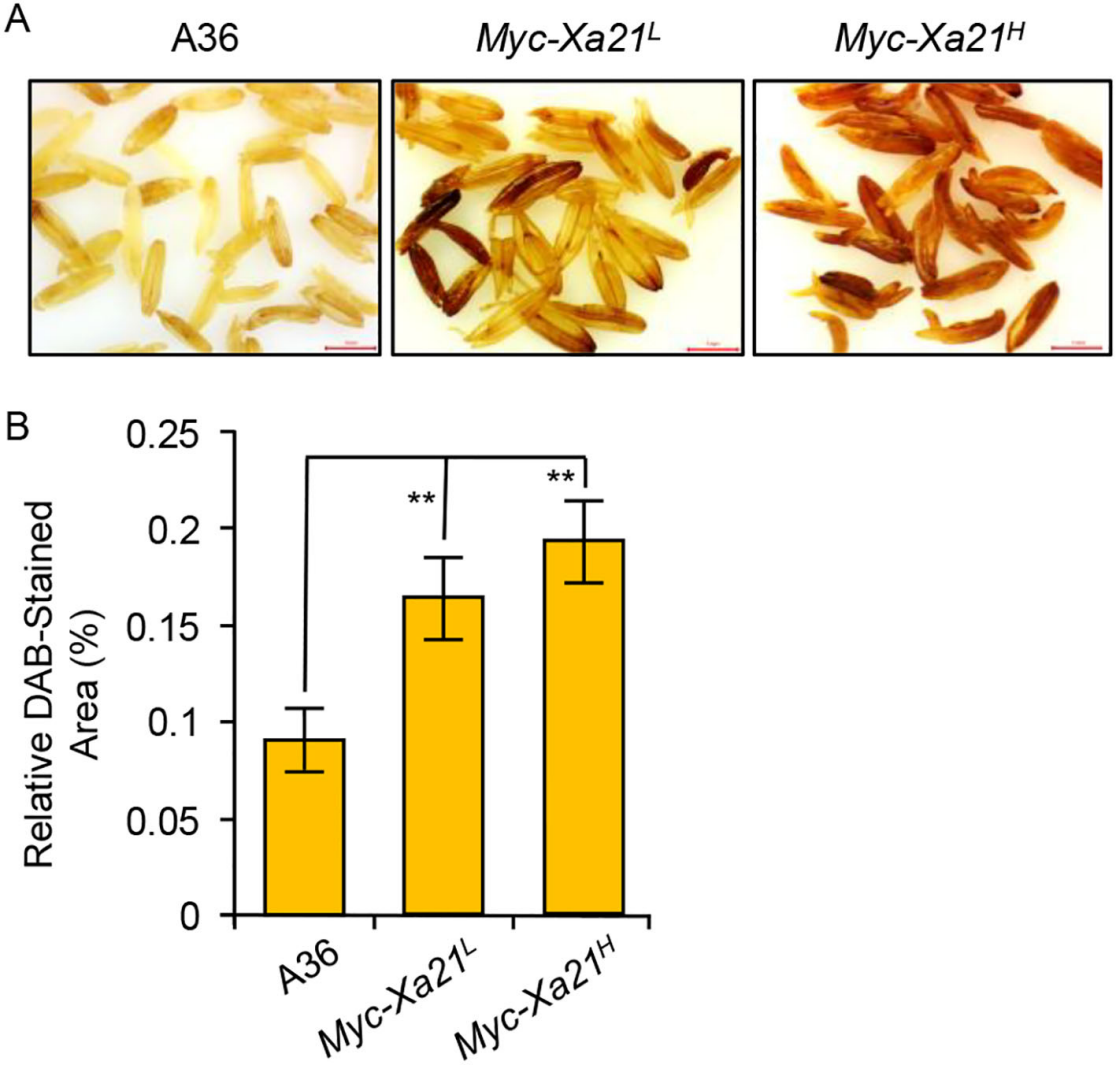
Reactive oxygen species (ROS) production in the anthers of *Myc-Xa21* plants at 24°C. **(A)** 3,3′-Diaminobenzidine (DAB) staining for H_2_O_2_ in anthers harvested at anthesis from A36 (empty vector control), *Myc-Xa21^L^*, and *Myc-Xa21^H^* plants grown. Samples were incubated with DAB solution overnight at room temperature. Scale bars, 1 mm. The experiments were repeated twice with similar results. **(B)** Quantification of ROS in the DAB-stained anthers of the indicated lines. Values are shown as the mean ± SD from seven anthers. Statistical analyses were performed using Student’s t-test. Asterisks denote statistically significant differences (**p < 0.01).

The treatment of rice leaves expressing XA21 with RaxX-sY induces the robust production of ROS (Pruitt et al., 2015, 2017; Chen et al., 2021). We examined ROS accumulation in the anthers of XA21-expressing lines and their control (A36) using DAB staining, an assay that mainly detects the accumulation of hydrogen peroxide in tissues (Daudi and O'Brien, 2012). As shown in **Figure 5**, increasing levels of oxidized DAB were visible in the *Myc-Xa21^L^* and *Myc-Xa21^H^* anthers at anthesis in the absence of *Xoo*. This finding indicates that ROS production is shared by the RaxX-sY-triggered XA21 defense response in the leaf and the XA21-dependent alterations in the anther.

## Discussion

*R* genes with high fitness costs in host plants rarely propagate/survive through evolutionary selection. However, increasing evidence indicates that plants have developed sophisticated mechanisms by which significant *R* genes are retained to fight against pathogen invasion. For instance, the *Cf-2* gene discovered in the wild tomato species *Lycopersicon pimpinellifolium* specifies resistance against the fungus *Cladosporium fulvum* (Dixon et al., 1996). In plants lacking a functional *Rcr3* gene (e.g., in the cultivated tomato *Lycopersicon esculentum*), which encodes a papain-like cysteine endoprotease, *Cf-2* activates autonecrosis (Kruger et al., 2002). The rice gene *PigmR* confers broad-spectrum resistance to *M. oryzae*, but its expression also causes a decrease in grain weight by approximately 2% (Deng et al., 2017). This detrimental effect is compensated for by *PigmS*, a gene located in the same locus (called *Pigm*), which is capable of increasing grain set by approximately 5%. Consequently, the *Pigm* locus with both *PigmR* and *PigmS* has been used for rice breeding for more than five decades without an overall yield penalty. We recently reported a rhomboid-controlled, post-translational regulatory mechanism through which rice prevents the over-accumulation of the R protein XA21, and potentially other transmembrane domain proteins, in spikelets to avoid fertility defects (Vergish et al., 2025).

We demonstrate here that XA21 decreases pollen viability and anther dehiscence. This study reveals the potent effect of temperature [24°C (daytime)] on the XA21 function. These findings are in line with our recent observation that an optimal daytime temperature (28 °C) facilitates the male sterility observed in the *osrbl3b Myc-Xa21^L^* mutant lines with a mutant rhomboid protease and an increased abundance of Myc-XA21 (Vergish et al., 2025) and with the previous discovery that a 4 °C decrease in temperature (from 31 °C to 27°C) fully activates the developmentally regulated *Xa21* resistance to the incompatible *Xoo* strain PXO99^A^ at the seedling stage (Chen et al., 2019). In contrast, high temperatures (31°C or above) can suppress *Xa21* functions (Chen et al., 2019; Vergish et al., 2025). As an intermediate level of resistance was observed at 29°C, we speculate that 24°C may not represent a “low temperature” threshold for XA21-induced fertility defects. Since its cloning in 1995 (Song et al., 1995), *Xa21* has been genetically engineered into various rice cultivars for the assessment of resistance and deleterious effects on plant growth, presumably under normal growth conditions. No developmental defects were reported in most of these studies (Li et al., 2001; Gao et al., 2013; Gayen et al., 2016; Zhang et al., 2024). Therefore, high-temperature tropical environments seem to mitigate the effects of XA21 on rice fertility. Since environmental conditions can greatly influence disease resistance in various plants (Cheng et al., 2019; Cohen and Leach, 2020), it is tempting to speculate that more examples of the costs associated with *R* genes may exist when plants are grown under distinct environments.

XA21-mediated fertility defects are dose-dependent and strongly modified by ambient temperature. Dependent on the low temperature, the *Myc-Xa21^H^* line (with a higher level of the XA21 protein) exhibits more severe fertility-related phenotypes than the *Myc-Xa21^L^* line. Independent of the temperature, the *osrbl3b-b Myc-Xa21^H^* mutant, which has even more abundant XA21 than *Myc-Xa21^H^*, is sterile (Vergish et al., 2025). These findings suggest a potential threshold of XA21 abundance that induces fertility defects. In an empirical field study, marked yield loss was observed in an *Xa21* transgenic line with high resistance (Hao et al., 2009). Such yield reduction could be attributed to a potentially very high level of *Xa21* expression (Zhang et al., 2024), although it is unclear whether temperature (e.g., growth seasons) may be another factor in this study. Notably, the highly resistant *Pi-d2* and *Pi-d3* lines also displayed yield penalties (Hao et al., 2009). *Pi-d2* and *Pi-d3* confer resistance to the fungus *M. oryzae* and encode an RLK with an extracellular domain of a bulb-type mannose-specific binding lectin and an *NLR* protein, respectively (Chen et al., 2006; Shang et al., 2009). Like *Xa21*, these genes, which encode distinct protein structures, may invoke the costs of fitness in a dose-dependent manner.

**Figure 6:**
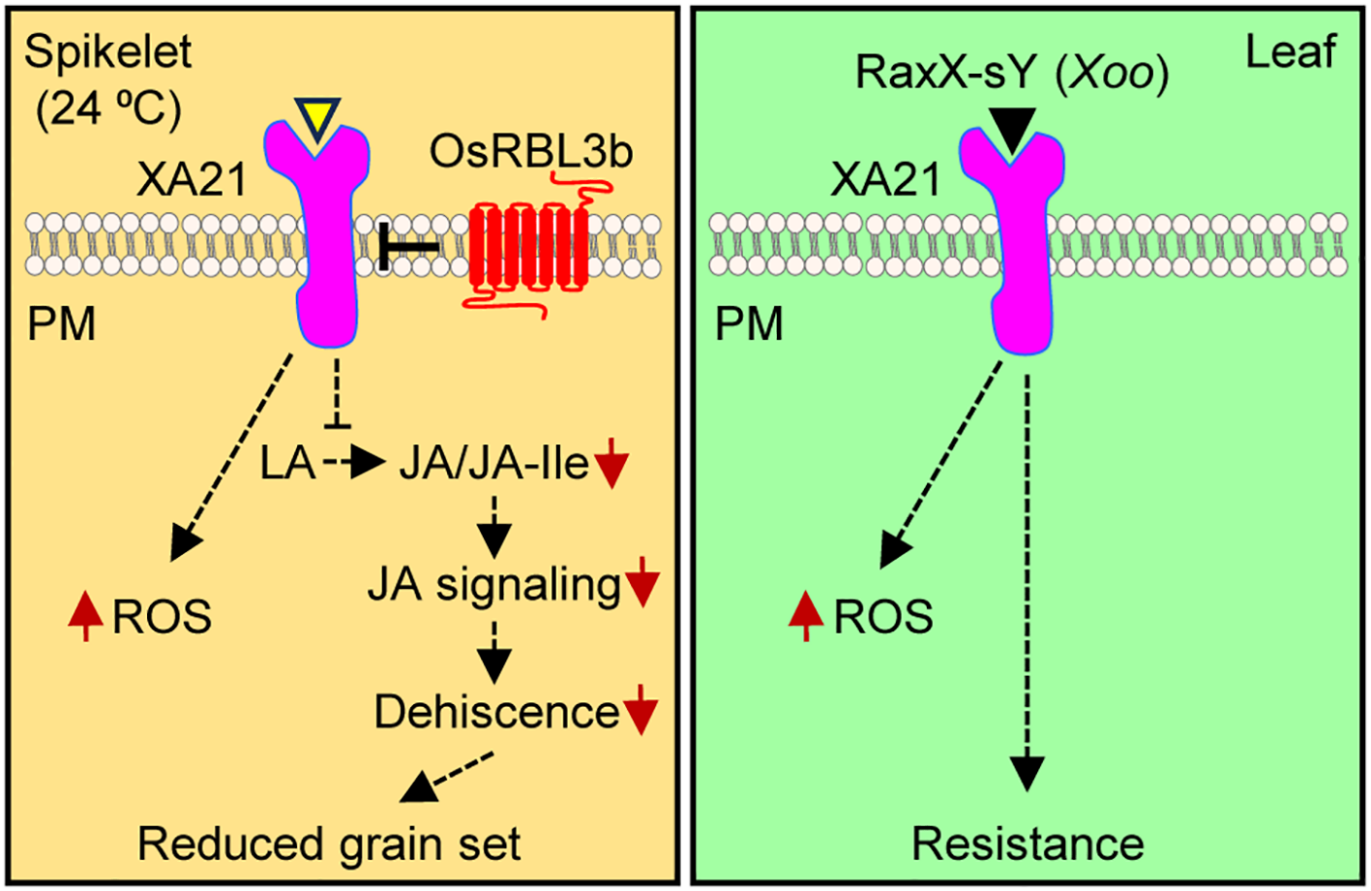
Simplified models of XA21-mediated signaling in spikelets and leaves. In spikelets during anthesis at 24°C, XA21 signaling is activated by an unidentified rice-derived signal (yellow triangle), leading to the production of reactive oxygen species (ROS) and to the suppression of jasmonic acid (JA) biosynthesis from its precursor linolenic acid (LA), which in turn represses JA signaling. Deceased JA signaling compromises anther dehiscence, ultimately resulting in reduced grain set. In leaves, XA21-mediated signaling is activated by the *Xoo* peptide RaxX-sY, which results in ROS production and resistance to *Xoo*. As described recently (Vergish et al., 2025), the rhomboid protease OsRBL3b prevents the over-accumulation of XA21 in rice spikelets.

To our knowledge, this work is the first to reveal the physiological and molecular mechanisms underlying *R* gene-instigated detrimental effects on reproduction. While the observed impairments to anther dehiscence and pollen viability can partially explain the semi-sterility of *Myc-Xa21* plants, the compromised JA signaling (**Figure 3**) could provide, at least in part, a molecular basis for *Xa21*-mediated male sterility (see **Figure 6** for a model). Among the 11 downregulated JA-responsive and JA-signaling genes in the semi-sterile line *Myc-Xa21^H^* at 24°C, four genes were also differentially regulated in the sterile line *osrbl3b-b Myc-Xa21^H^* under greenhouse conditions during the summer (**Figure 3**; Vergish et al., 2025), suggesting that altered JA signaling may have a role in the fertility defects of both *Myc-Xa21^H^* and *osrbl3b-b Myc-Xa21^H^*. Furthermore, the sharp enrichment of differentially downregulated JA biosynthesis genes in *Myc-Xa21^H^* in this study suggests that XA21 signaling mediates the downregulation of JA responsiveness, which may lead to fertility defects (**Figure 6**). This hypothesis is supported by our observations that JA and JA-Ile contents were decreased in *Myc-Xa21^H^* spikelets during anthesis relative to the control (A36) and that exogenous treatment with MeJA was capable of restoring *Myc-Xa21* anther dehiscence at 24°C. Further support of the hypothesis comes from the promoter GUS reporter assay, in which the *Xa21* promoter was found to be active in the anther filaments, the veins of the palea/lemma, and the rachillae of the spikelets (**Figure 4**). Consistently, the *Arabidopsis Defective in Anther Dehiscence* (*DAD1*) gene, key to JA biosynthesis and anther dehiscence, is exclusively expressed in anther filaments before anthesis (Ishiguro et al., 2001). It has been hypothesized that JA accumulation in anther filaments promotes water movement from anther locules to the filaments. This dehydration of anther locules facilitates the maturation of pollen grains and induces anther dehiscence (Ishiguro et al., 2001; Acosta and Przybyl, 2019). In addition, transgenic rice lines ectopically expressing *Xa21* from the constitutive maize ubiquitin promoter show very high levels of XA21 and resistance to *Xoo* in the leaf but normal grain set (Park et al., 2010b), indicating the need for the native promoter to condition *Xa21* costs. A related finding is our discovery that the exogenous application of JA compromises XA21-mediated resistance to *Xoo* at the 2-week-old seedling stage (Chen et al., 2025), suggesting that XA21 function and JA signaling interact antagonistically in rice.

It may be worth noting that the signaling mechanisms in the semi-sterile line *Myc-Xa21^H^* at 24°C and the sterile mutant *osrbl3b-b Myc-Xa21^H^* at high temperatures (greenhouse conditions) may differ from each other. Unlike *Myc-Xa21^H^*, no significant enrichment of the altered expression of JA biosynthesis genes was observed in the sterile *osrbl3b-b Myc-Xa21^H^* spikelets compared to the fertile *Myc-Xa21^H^* spikelets at anthesis at high temperatures (Vergish et al., 2025). In contrast to the *osrbl3b-b Myc-Xa21^H^* mutant, the enrichment of the upregulated expression of *NLR* genes is not significant in *Myc-Xa21^H^* (**Supplementary Figure S3**; Vergish et al., 2025). These discrepancies may be due to the potential cleavage of additional signaling regulators by OsRBL3b (Vergish et al., 2025) and the suppression effects of elevated temperatures on XA21 signaling (Chen et al., 2019), which collectively lead to the activation of distinct signaling pathways in the *osrbl3b-b Myc-Xa21^H^* mutants.

The increased accumulation of ROS in spikelets may also contribute to *Xa21*-mediated male sterility. During anther development, proper levels of ROS trigger tapetal degradation, which provides nutrients for pollen (Zhang et al., 2021; Xie et al., 2022). However, the abnormal accumulation of ROS can damage tapetal function and pollen development, leading to male sterility. XA21-mediated fertility defects may be caused by the activation of the receptor by an endogenous signal in rice spikelets (**Figure 6**). Previous studies have shown that neither low-temperature treatment nor abundant XA21 produced from ectopic expression by the strong maize ubiquitin promoter can activate the receptor, as XA21 plants are susceptible to the compatible *Xoo* strains under the above conditions (Park et al., 2010b; Chen et al., 2019; Zhang et al., 2024). Given that ROS production in XA21 leaves is triggered by the *Xoo* peptide RaxX-sY (Pruitt et al., 2015; Chen et al., 2021), our observation of XA21-dependent ROS accumulation in anthers at 24°C suggests that the receptor may be activated by an endogenous signal, although the impact of abundant ROS on grain development remains to the determined. Low temperature (24°C) treatment here likely primes the activation of XA21 signaling, as suggested previously (Chen et al., 2019). XA21 is among a set of immune receptors that are present in both plants and animals (Ronald and Beutler, 2010; Ercoli et al., 2022). Autoimmunity and autoimmune diseases (e.g., type I diabetes) have emerged as a worldwide threat to public health (e.g., estimated yearly increase in the prevalence of human autoimmune diseases is 12.5%) (Miller, 2023). Current views underscore the importance of environmental cues (including temperature) and genetic risk factors for the rise of autoimmune disorders; however, mechanistic insights remain elusive (Miller, 2023). Further studies may lead to a deeper understanding of autoimmunity and to the development of strategies to control such disorders.

In the 1990s, we identified a total of 17 transposable-like elements (TEs) in the *Xa21* locus, which harbors at least six *Xa21*-related family members originating from *O. longistaminata*, with member D being 98% identical to *Xa21* at the DNA level (Song et al., 1997, 1998). Interestingly, D provides partial resistance to the same spectrum of *Xoo* strains as *Xa21* does, likely because it is compromised by insertion of a retrotransposon (called *Retrofit*) into its coding region (Wang et al., 1998). At the time, the selective advantage of the accumulation and movement of these TEs was unclear (Song et al., 1998). Our findings in this study suggest that the TEs may represent a genomic tool by which rice controls the deleterious function of *Xa21* and possibly member D. This idea is in line with the prevalent hypothesis, proposed by Barbara McClintock and others, that TEs may facilitate genome responses to challenges (McClintock, 1984; Wessler, 1996; Slotkin and Martienssen, 2007).

## Data Availability

The original contributions presented in the study are publicly available. This data can be found here: NCBI, PRJEB102018.

## Supplementary material

Supplementary Figure 1 *Xa21* decreases grain-set in *Oryza sativa* ssp. *japonica* variety Kitaake at 24 °C. **(A)** Panicle phenotypes of the rice lines Kitaake (wild-type control) and 20-1 (also called 20-1-18–1 or 18–1 in the lab) that expresses *Myc-Xa21* under its native promoter. **(B)** Phenotypes of mature plants of the indicated lines in **(A)**. **(C)** Grain-setting rates of plants in **(A)**. Values are shown as the mean ± SD from 5 independent plants with top two panicles each. Statistical analyses were performed using Student’s *t*-test. Asterisks denote statistically significant differences (***P* < 0.01). **(D)** Dehusked grains harvested from the indicated lines in **(A)** showing the difference in grain set between these two lines.

Supplementary Figure 2 Mass spectrometry identification of jasmonic acid (JA) and JA-isoleucine (JA-Ile). In each panel, the top MS/MS spectrum in blue represents the authentic standard spectrum, and the bottom spectrum in purple is from the samples.

Supplementary Figure 3 RNA-seq volcano plot showing differentially expressed rice *NLR* genes. A complete list of transcript IDs detected is shown previously (Vergish et al., 2025). Cut-off criteria for data: log_2_[*Myc-Xa21^H/^*A36] ≥ 1 and ≤ -1 and *FDR*-q value < 0.05.
