# Supplementary figures and images for: The immune receptor XA21 causes semi-male sterility and grain loss in rice
Source: Front Plant Sci. 2025 Nov 10;16:1673821. doi: 10.3389/fpls.2025.1673821 (PMC12640976; doi:10.3389/fpls.2025.1673821)

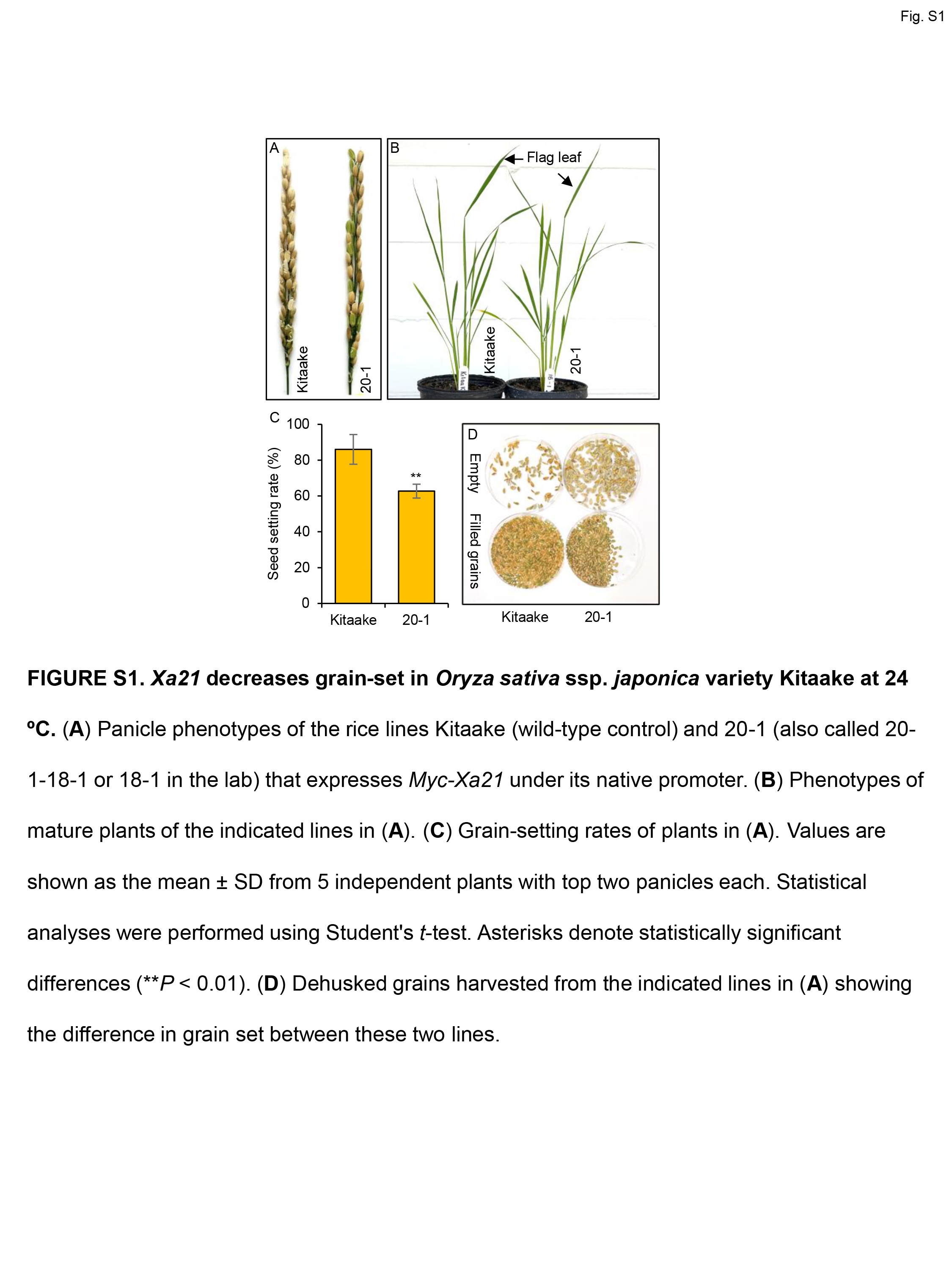

Supplement: Supplementary Figure 1 — Xa21 decreases grain-set in Oryza sativa ssp. japonica variety Kitaake at 24 °C. (A) Panicle phenotypes of the rice lines Kitaake (wild-type control) and 20-1 (also called 20-1-18–1 or 18–1 in the lab) that expresses Myc-Xa21 under its native promoter. (B) Phenotypes of mature plants of the indicated lines in (A). (C) Grain-setting rates of plants in (A). Values are shown as the mean ± SD from 5 independent plants with top two panicles each. Statistical analyses were performed using Student’s t-test. Asterisks denote statistically significant differences (**P < 0.01). (D) Dehusked grains harvested from the indicated lines in (A) showing the difference in grain set between these two lines. [file Image1.tif]

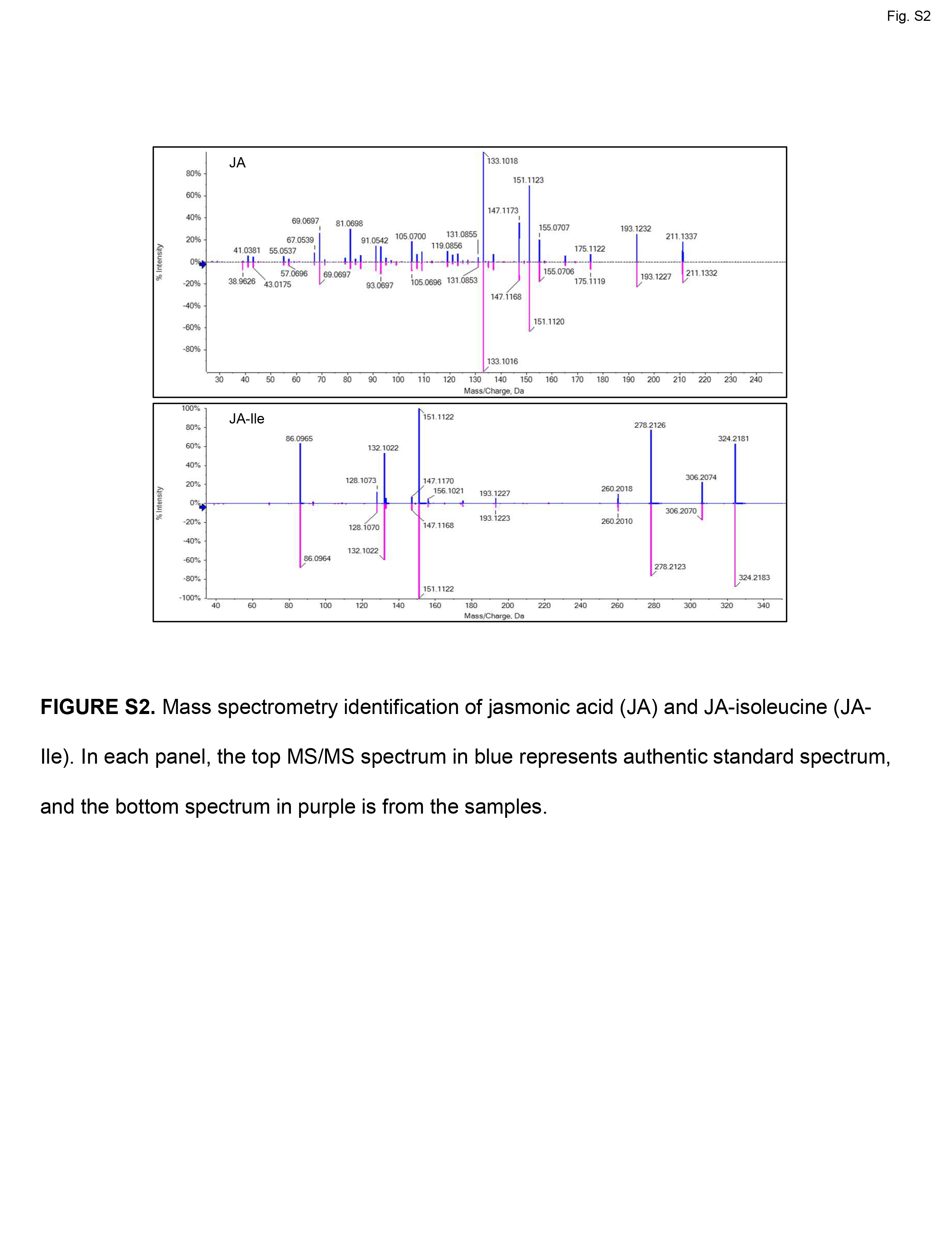

Supplement: Supplementary Figure 2 — Mass spectrometry identification of jasmonic acid (JA) and JA-isoleucine (JA-Ile). In each panel, the top MS/MS spectrum in blue represents the authentic standard spectrum, and the bottom spectrum in purple is from the samples. [file Image2.tif]

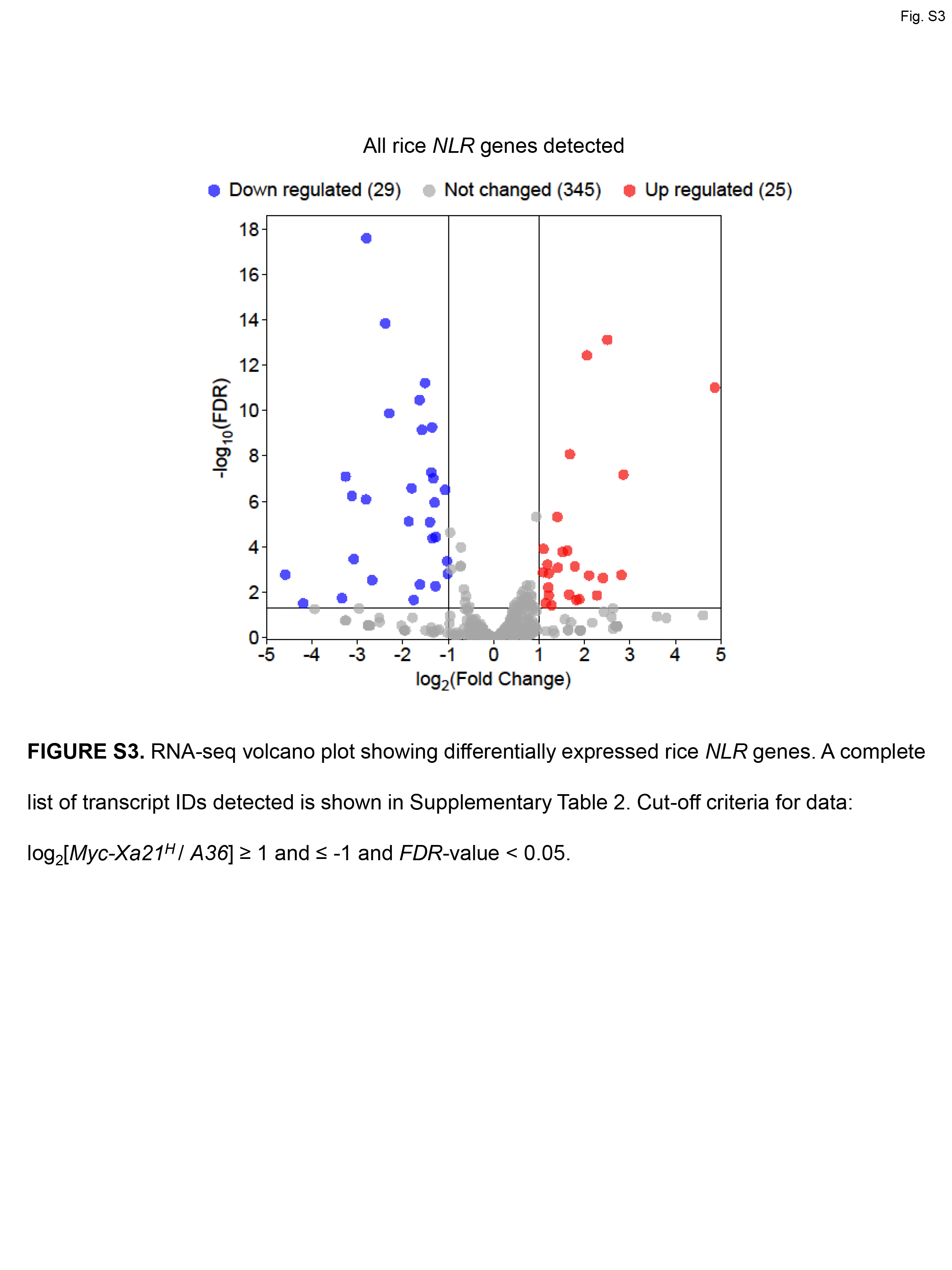

Supplement: Supplementary Figure 3 — RNA-seq volcano plot showing differentially expressed rice NLR genes. A complete list of transcript IDs detected is shown previously (Vergish et al., 2025). Cut-off criteria for data: log2[Myc-Xa21H/A36] ≥ 1 and ≤ -1 and FDR-q value < 0.05. [file Image3.tif]
